# INFLAMM-AGING IS ASSOCIATED WITH PRO-INFLAMMATORY PROGRAMMING OF INNATE IMMUNE CELLS IN THE COLON

**DOI:** 10.1093/geroni/igac059.1734

**Published:** 2022-12-20

**Authors:** Ji Yeon Noh, Hongying Wang, Yuhua Farnell, Xiao-Di Tan, Gus Wright, Andrew Hillhouse, Yuxiang Sun

**Affiliations:** Texas A&M University, College Station, Texas, United States; Texas A&M University, College Station, Texas, United States; Texas A&M AgriLife Research, Texas A&M University, College Station, Texas, United States; Ann & Robert H. Lurie Children’s Hospital of Chicago, Feinberg School of Medicine, Northwestern University, Chicago, Illinois, United States; College of Veterinary Medicine & Biomedical Sciences, Texas A&M University, College Station, Texas, United States; College of Veterinary Medicine & Biomedical Sciences, Texas A&M University, College Station, Texas, United States; Texas A&M University, College Station, Texas, United States

## Abstract

Chronic low-grade inflammation is prevalent in aging, which is called inflamm-aging. Immune cells are important mediator of inflammatory state of host and the innate immunity is the first responder to various insults. Gastrointestinal track, especially colon, is the site where immune cells are abundant. Aging is associated with increased gut dysbiosis and functional decline. We hypothesize that colonic innate immune cells contribute to the age-associated inflammation in the colon. We found that macrophages in colon mucosa are elevated in aging, and it is accompanied by pro-inflammatory cytokine expression and increased gut permeability. Specifically, we used flow cytometry to assess colonic innate immune cells collected from 5-, 7-, 14-, 19-, 24-, and 28-month-old mice. Aging significantly increased the populations of pro-inflammatory cytokine producing innate immune cells in the colon, including neutrophils, dendritic cells, monocytes and macrophages. Interestingly, the infiltrating Ly6Chi macrophages and pro-inflammatory CD11c+ macrophages were much higher, while anti-inflammatory CD206+ macrophages were decreased in colon of the aged mice. In line with the immune profile, aged mice showed increased gut permeability tested by fluorescein isothiocyanate-dextran, and the gene expressions of gap junction proteins in the colon were decreased, supporting increased gut permeability in the aged mice. Collectively, our results suggest that innate immune cells play an importance role in age-associated gut inflammation; targeting the innate immune cells in the colon may present a novel therapeutic strategy for prevention and treatment of aging leaky gut.

